# Leptomeningeal Carcinomatosis: A Case Report of Metastatic Triple-negative Breast Adenocarcinoma

**DOI:** 10.7759/cureus.4278

**Published:** 2019-03-19

**Authors:** Nathaniel Parker, John Forge, Daniel Lalich

**Affiliations:** 1 Internal Medicine, University of Kansas School of Medicine, Wichita, USA; 2 Pathology, Wesley Medical Center, Wichita, USA

**Keywords:** leptomeningeal carcinomatosis, leptomeningeal metastasis, leptomeningeal disease, brain tumor, breast cancer, triple-negative breast adenocarcinoma, late-stage complication

## Abstract

A 55-year-old female presented to the emergency department with seizures, left hemiparesis, and memory loss. Her past medical history was notable for a right triple-negative breast adenocarcinoma that was diagnosed approximately two years prior. She underwent treatment with chemotherapy, right breast lumpectomy, and radiation near her rural hometown. Radiologic studies were performed in the emergency department. Brain imaging revealed a new 2-cm mass in the left breast and a 4-cm left frontal lobe brain lesion. She underwent an urgent craniotomy. Immunohistochemical staining of the brain tumor tissue suggested metastatic triple-negative breast adenocarcinoma. She was discharged with recommendations to follow up with her prior oncologist near her home for systemic chemotherapy. Three months after metastatic breast cancer to the brain was diagnosed, the patient experienced headaches, fever, and nuchal rigidity. MRI of the brain showed new leptomeningeal enhancement. A lumbar puncture with a cerebrospinal fluid analysis revealed the presence of malignant cells. Together with imaging and cerebrospinal fluid findings, leptomeningeal carcinomatosis was diagnosed. This case report presents an uncommon but well-known complication of breast cancer.

## Introduction

Leptomeningeal carcinomatosis (LC) is a late-stage complication of malignant tumors that metastasize to the cerebrospinal fluid (CSF) and leptomeninges. The clinical presentation of LC is variable, as any level of the central nervous system (CNS) may be affected [1]. Patients most commonly report migraines, altered mentation, cerebellar signs, back pain, and leg weakness at the time of initial presentation [2]. Also, leptomeningeal tumor growth can impair CSF flow and lead to symptoms of hydrocephalus, like positional headaches, nausea, vomiting, and somnolence [3]. The incidence of LC is challenging to determine [3]. Based on autopsy studies of cancer patients who had neurologic symptoms, the prevalence of leptomeningeal disease is approximately 19% [1]. LC has become increasingly reported in living cancer patients. It is clinically recognized in up to 8% of all cancer patients [3]. Explanations for more recent reports of LC exist. Improved neuroimaging methods, treatments extending the survival of cancer patients, and the use of systemic anti-cancer agents that do not cross the blood-brain barrier may play a vital role [4-6].

## Case presentation

A 55-year-old female nonsmoker presented to the emergency department with progressively worsening seizures, left hemiparesis, and memory loss. Two years prior, she was diagnosed with right-breast triple-negative adenocarcinoma (TNBC). Her treatment regimen was as follows: 1.) neoadjuvant chemotherapy with doxorubicin, cyclophosphamide, and paclitaxel; 2.) right breast-conserving surgery by lumpectomy; and 3.) postoperative radiation. Overall, she completed 35 cycles of chemotherapy and three months of radiation. Her last documented therapy was administered approximately one year prior to the initial patient encounter, and her breast cancer was thought to be in remission.

**Figure 1:**
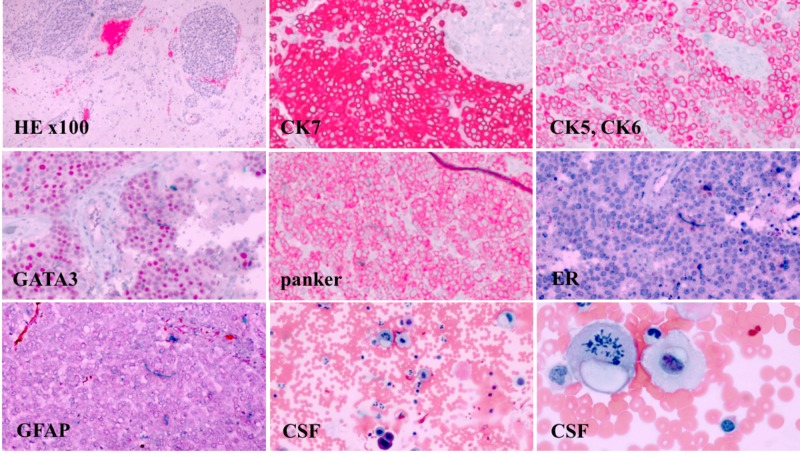
The pathology specimen demonstrates poorly differentiated metastatic carcinoma of the breast. (H&E stain, x100). Brain tissue shows carcinoma cells are positive for CK7, CK5, CK6, GATA3, and panker, but negative for ER and GFAP. PR and Her2/neu IHC staining performed at a specialty facility, and slides could not be obtained but reported as negative. Neoplastic cells in the CSF with pleomorphic nuclei and prominent nucleoli (Wright-Giemsa stain x100). Higher-power magnification shows carcinoma cells with cytoplasmic vacuoles and mitotic figures (Wright-Giemsa stain x400). H&E: hematoxylin and eosin; CK: cytokeratin; GATA binding protein 3; ER: estrogen receptor; GFAP: glial fibrillary acidic protein; Her2/neu: human epidermal growth factor receptor 2/neu; IHC: Immunohistochemistry; CSF: cerebrospinal fluid

In the emergency department, computed tomography (CT) scans of the head and magnetic resonance imaging (MRI) of the brain with contrast were obtained. MRI showed a large right frontal lobe lesion measuring approximately 4 cm with surrounding hemorrhagic necrosis. Additionally, a 1.2 cm right to left midline shift anteriorly, effacement of the anterior right lateral ventricle, mass effect, and herniation were evident. No leptomeningeal enhancement was noted. CT scans of the chest also showed a new 2-cm mass in the left breast. There was no CT evidence of lymphadenopathy or metastasis in the chest. Regional skeletal tissue changes were age-appropriate and without focal lytic or blastic lesions. Image findings, in conjunction with the patient’s clinical picture, were concerning for the development of metastatic central nervous system (CNS) disease. A frontal craniotomy was performed in an effort at tumor resection and helped provide a final pathological diagnosis. Properly controlled immunohistochemical stains for cytokeratin 7 (CK7), cytokeratin 5 and 6 (CK5, CK6), gross cystic disease fluid protein 15 (GCDFP-15), GATA binding protein 3 (GATA3), pan-cytokeratin (panker), estrogen receptor (ER), progesterone receptor (PR), human epidermal growth factor receptor 2/neu (HER2/neu), and glial fibrillary acidic protein (GFAP) were performed on brain tumor tissue. Malignant-appearing cells were noted and exhibited positive immunoreactivity for CK7, CK5-6, GCDFP-15, GATA3, and pan-cytokeratin. Tumor cells were negative for ER, PR, and HER2/neu (Figure 1). This IHC staining profile supported metastatic TNBC.

The patient tolerated craniotomy well and her postoperative period was unremarkable. Her neurologic symptoms improved with anti-epileptic medications. She was discharged with recommendations to follow up with her prior oncologist near her home for systemic chemotherapy. Records lacked information regarding left breast lumpectomy. Also, there was no record of BRCA genotyping at the time of the right and left breast cancer diagnosis.

Two months after craniotomy, CT scans of the chest, abdomen, and pelvis were unremarkable for metastatic disease. However, subsequent positron emission tomography-CT demonstrated a small nodular area of fluorodeoxyglucose (FDG) activity in the right frontal lobe, suggesting possible tumor regrowth. Expected underlying right frontal lobe post-craniotomy changes were present. No additional FDG-avid lesions were identified. She underwent localized non-surgical, high precision stereotactic radiosurgery (SRS) to this region and the right frontal lobe post-craniotomy operative bed for the preventative management of brain tumor regrowth. She received a total of three SRS treatments to the right frontal brain lobe.

Three months following craniotomy, she presented to the emergency department with severe headaches, high-grade fevers, and nuchal rigidity. Physical exam was primarily benign. The workup was negative for infectious etiologies. Given the patient’s clinical history and new neurologic symptoms, concerns for leptomeningeal disease were raised. Brain and whole-spine MRI was performed, which revealed abnormal enhancement in the brain and spinal T6-8 meningeal enhancement. Lumbar puncture with CSF analysis was performed, which showed the presence of large atypical cells suggestive of metastatic adenocarcinoma (Figure 1). Together with imaging and CSF analysis, LC was diagnosed. Treatment was palliative with intrathecal methotrexate (IT MTX) to improve her neurologic symptoms and prolong survival. However, she experienced a decline of sensorium and functional status despite three weeks of IT MTX. Subsequent lumbar punctures with CSF analysis showed the persistence of malignant cells. Also, repeat MRI of the brain showed new metastatic disease to the cerebellum, leptomeningeal enhancement in the right temporal lobe, and significant ventriculomegaly. IT MTX therapy was stopped, and she was transitioned to hospice care.

## Discussion

Leptomeningeal metastasis is an uncommon but classic late-stage complication of breast cancer that has become increasingly prevalent. Breast cancer, non-small-cell lung cancer, and melanoma most commonly metastasize to the leptomeninges [3]. Furthermore, adenocarcinomas from any primary site are the most common histologic tumor type to metastasize to the leptomeninges [3]. Breast cancer consistently contributes the most to the total leptomeningeal disease population despite only about 5% of breast cancer patients developing leptomeningeal involvement [1,3]. Initial physical exam findings may be subtle or isolated and ultimately overlooked [3]. Clinical suspicions must remain high in known metastatic cancer patients that develop neurological symptoms [7]. A thorough clinical history is vital. LC must be differentiated from other entities that can present with neurological manifestations, such as infectious meningitis, metabolic and toxic encephalopathies, sarcoidosis, paraneoplastic syndromes, or chemoradiation side effects [1]. Increased clinical awareness of LC allows for earlier detection and treatment, maintains the quality of life, and prolongs survival [1].

MRI is critical in the initial workup process for leptomeningeal diseases. Since these metastatic malignancies can involve the entire CNS, brain with whole spine imaging is recommended [8]. T1 and T2-weighted sequences with contrast should be performed. Differentiating metastatic leptomeningeal enhancement from CNS vasculature or flow artifacts is often challenging if MRI is not done in enough enhanced planes [9]. Evaluation and clinical decision-making based on CT or myelography are not recommended [8]. These imaging studies have a significantly lower sensitivity as compared to MRI [8]. The characteristic finding of leptomeningeal involvement on MRI is meningeal enhancement [9]. Radiologically, metastatic neoplasms to the leptomeninges are more evident at the base of the brain, dorsal spinal cord, and cauda equina [1].

The gold standard for the diagnosis of metastatic leptomeningeal disease is the presence of malignant cells in CSF [10]. However, significant false-negative rates have been reported [5]. Initial negative cytology rates have reached as high as 50% in observational studies [9]. Thus, serial CSF sampling can improve sensitivity [5]. However, a retrospective analysis of 200 leptomeningeal disease patients revealed that 53% of patients were diagnosed by MRI, 23% by cytology, and 24% by both methods [11]. MRI findings are typically only abnormal in 75%-90% of patients with cytology-positive CSF [5]. Ultimately, experts agree that neither MRI nor CSF sampling is a sensitive enough method when used alone [11]. Thus, a suggestive clinical picture together with either MRI findings or a CSF analysis is sufficient for conditions such as LC [5].

Despite advancements in imaging and therapeutic interventions, LC remains a classic and well-known complication of breast cancer. Median interval times from the determination of the initial primary solid tumor to leptomeningeal involvement is approximately 24 months [12]. TNBC status has a poorer prognosis as compared to hormone-sensitive or HER2-positive breast cancer [13]. TNBC has a higher likelihood of later leptomeningeal involvement [12]. Moreover, TNBC is associated with a shorter interval between initial primary breast cancer diagnosis and the development of leptomeningeal disease [12]. Once LC has been confirmed, treatment options remain sparse and survival is often short [14]. The standard treatment for LC is IT MTX plus systemic chemotherapy with or without localized radiation therapy [3]. Due to these treatment modalities, median survival has improved from about one month to three to six months [3]. Radiotherapy can be useful for resolving impaired CSF flow in bulky leptomeningeal disease [3,15]. However, metastatic leptomeningeal tumors rarely progress to localized bulky disease [3,15]. IT trastuzumab for HER2-positive disease has shown to be effective [15]. More strategies using other IT chemotherapy agents for leptomeningeal disease regardless of ER/PR/HER2 status have been investigated [16]. However, IT chemotherapy has its limitations. Besides toxicity, IT chemotherapy efficacy is CSF flow-dependent [17]. Approximately, 50% of patients with leptomeningeal disease have evidence of CSF flow obstruction. Thus, intracranial pressure measurements before administration are recommended. However, no current treatment modality has been shown to improve overall survival [17].

## Conclusions

LC is an uncommon but well-known oncological entity that represents a terminal complication of breast cancer. TNBC has a higher likelihood of leptomeningeal metastasis. Clinical suspicions should remain high in patients with a positive breast cancer history who present with focal neurological signs despite a history of prior breast cancer treatment. Further research is needed to determine better treatment options.
